# Linking pod-set and seed yield of faba bean across organ, phytomer, plant, and population scales

**DOI:** 10.1093/jxb/eraf176

**Published:** 2025-05-04

**Authors:** James B Manson, Matthew D Denton, Lachlan Lake, Jason Brand, Victor O Sadras

**Affiliations:** School of Agriculture, Food and Wine, The University of Adelaide, Adelaide, Australia; School of Agriculture, Food and Wine, The University of Adelaide, Adelaide, Australia; School of Agriculture, Food and Wine, The University of Adelaide, Adelaide, Australia; South Australian Research and Development Institute, Adelaide, Australia; College of Science and Engineering, Flinders University, Adelaide, Australia; Frontier Farming Systems, Australia; School of Agriculture, Food and Wine, The University of Adelaide, Adelaide, Australia; South Australian Research and Development Institute, Adelaide, Australia; College of Science and Engineering, Flinders University, Adelaide, Australia; University of Cambridge, UK

**Keywords:** Competition, dominance, flower overproduction, grain legume, phenotype, plant population density, pod-set, seed size, trade-off, *Vicia faba*

## Abstract

Pod-set is the conversion of flowers to pods, but its connection to crop yield of pulses must traverse scales of biological organization; here we address scaling from organ to crop in faba bean. We hypothesized that crop-level resource capture drives pod-set and yield of faba bean, whereas allocation to pods or between pods plays a minor role. We combined new field experiments and published data to test supporting hypotheses in four studies. We showed that node-level pod-set is not modular but is coordinated across the whole plant. Crop yield and pods per m^2^ were associated with crop growth in our data, not with plant growth or supposed competition between plants or pods. Seed number, seed size, and pod wall mass had yield-neutral trade-offs. Indeterminate shoots facilitate resource capture, and, surprisingly, fruit removal could increase yield by up to 49% when it allows continued growth and resource capture. We concluded that pod-set is mainly dependent on crop growth but could be targeted for its feedback to post-flowering phase duration. Our work provides conceptual links between plant reproductive biology and crop yield that could be relevant to other indeterminate crop species.

## Teaser

Faba bean overproduces flowers and serially aborts them to a final pod number. We show that final pod number depends on resource availability, and flower abortion is neutral for yield.

## Introduction

Pod-set is the successful conversion of flowers to mature, seed-bearing pods, and a low flower:pod ratio is often bemoaned as an obstacle to higher crop yields in pulses (Patrick and Stoddard, 2010). However, overproduction of flowers is nearly universal in plants (Stephenson, 1981), and changes to yield components are prone to trade-offs (Reynolds *et al.*, 2022). Furthermore, the contemporary definition of crop yield is mass of grain produced per area, and as such it is a population trait (Cossani and Sadras, 2021). Low plant population density increases pod-set per plant but can jeopardize crop-level growth and yield (Postma *et al.*, 2021; Manson *et al.*, 2024b). Before pursuing increased pod-set, a broader, integrated perspective is needed to connect it to the phenotype across scales of organization, from pod (organ), to node (phytomer), to plant (individual), to crop (population).

Here we address the scaling from organ to population with a focus on faba bean (*Vicia faba* L.), an under-researched winter pulse crop grown on ~2.75 Mha globally (Manners and van Etten, 2018; FAOSTAT, https://www.fao.org/faostat/). Yield is strongly associated with pod number (Loss and Siddique, 1997; López-Bellido *et al.*, 2005; Manson *et al.*, 2024a), and the flower-to-pod ratio varies from 6% to 60% (Kambal, 1969; Alharbi and Adhikari, 2020). Several attempts to increase pod retention with a focus on the organ scale have been unsuccessful or inconsistent, including selection for determinacy (Patrick and Stoddard, 2010), independent vascular structure of racemes (Ruckenbauer and Mollenkope, 1986), pollen supplementation (Bishop *et al.*, 2020), and application of plant hormones (Rylott and Smith, 1990). Faba bean studies have partially addressed multiple scales but did not engage with current physiological theory of yield determination (López-Bellido *et al.*, 2005; Patrick and Stoddard, 2010; Alharbi and Adhikari, 2020; Mínguez and Rubiales, 2021).

In most crop species, seed size is highly heritable and stable across environments, so crops accommodate variation in resources largely through a high plasticity in seed number (Sadras, 2007; Sadras and Slafer, 2012; Slafer *et al.*, 2014). This conforms to the classic Smith–Fretwell model that predicts that if the probability of survival of an offspring has an attenuating response to its size, then it is in the interest of the mother to target a size for each offspring that is adequate for survival, then allocate additional resources to make additional offspring (Smith and Fretwell, 1974). A highly heritable and stable seed size appears to also be a characteristic of faba bean, but published experiments include limited environments; a larger dataset would confirm the relevance of the model (Toker, 2004; Manson *et al.*, 2024a). The number of seeds per pod is highly heritable and stable, so here we consider pod and seed number as interchangeable traits that describe how faba bean accommodates variation in resources (Thompson and Taylor, 1977; Hebblethwaite *et al.*, 1984; Bond *et al.*, 1985; Loss and Siddique, 1997; Sau and Mínguez, 2000; Manson *et al.*, 2024a).

Seed number is determined during a species-specific ‘critical period’ (Carrera *et al.*, 2024). Growth during the critical period has a disproportionate effect on seed yield, and this underpins the general but less robust association of total growth and yield (Egli, 2011; Slafer *et al.*, 2023). In common with other indeterminate species, the critical period of faba bean, as determined by the standard experiment of sequential shading treatments, is after flowering, centred on the first pods visible (Lake *et al.*, 2019). Reinforcing this, pod number, seed number, and yield, but not seed size, are associated with growth during the critical period (Lake *et al.*, 2019; Manson *et al.*, 2024a).

For a given amount of growth in the critical period, genetic variation in seed size will trade-off with seed number (Sadras, 2007; Manson *et al.*, 2024a). If there are no confounding factors, the trade-off will be neutral for yield; this is a testable hypothesis: for a given amount of resources, the relationship between seed number and size will have a slope of –1 on a log–log scale (Henery and Westoby, 2001). If seed size or number are confounded with traits such as the accessory costs of packing seeds in pods, the slope of the relationship could deviate from –1 and it would be possible to target pod and seed number independently to change yield. This is also testable: the relationship between seed size and accessory cost per seed has a slope of +1 on a log–log scale (Henery and Westoby, 2001). The hypotheses were confirmed for 47 species of Australian perennial shrubs and trees (Henery and Westoby, 2001), although the approach has not been applied to grain crops. If confirmed, selection for increased pod-set would be neutral for yield, all else being equal, suggesting that pod number is merely a component of yield, not a determinant.

During the critical period, flowers are overproduced and sequentially aborted to determine a final seed number in response to resource availability, stress, and non-resource signals such as daylength (Lloyd, 1980; Ghiglione *et al.*, 2008; Sadras and Slafer, 2012; Sadras and Dreccer, 2015; Nico *et al.*, 2016), so we consider pod number more important than flower-to-pod ratio. Flower overproduction provides plants with many, well-documented benefits, several of which are related to pollination (Haig and Westoby, 1988; Burd and Callahan, 2000; Holland and Chamberlain, 2007; Sakai, 2007). Faba bean has a mixed-mating system with genotypic variation reliant on pollinators; pollination rates and the yield response to pollen vary between genotypes, environments, and experimental methods (Bishop *et al.*, 2020; Brünjes and Link, 2021; Adhikari *et al.*, 2023).

After flower production and abortion, the distribution of pods along a faba bean stem is lanceolate, with higher pod-set on basal nodes. This has been explained by temporal gradients in the environment and light intensity at the leaf of the given node (Ishag, 1973; Stoddard, 1993a; Manning *et al.*, 2024), implying that pod-set occurs at each node as a distinct event. It has also been explained as the result of pods competing for resources with stems or with each other, through either direct pre-emption of resources or hormonal signalling (Diethelm *et al.*, 1988; Ravishankar *et al.*, 1995), implying processes occurring between nodes. A key question is: to what extent are faba bean nodes modular during pod-set, where ‘modularity’ describes a high degree of phenotypic integration within a unit of a plant that is uncoupled from other units (Wesselingh, 2007; Diggle, 2014). Furthermore, the lanceolate distribution of pods is present in all faba bean datasets across environments, genotypes, and treatments, suggesting a lack of variation for yield improvement. A second key question is: is variation in the lanceolate distribution of pods associated with yield?

As an indeterminate species, faba bean continues to produce leaf-bearing nodes during reproduction. Early sowing and irrigation before flowering have been associated with increased vegetative biomass and reduced pod-set, but the net effect on yield can be positive or negative (Manson *et al.*, 2025). In principle, inhibiting vegetative growth would free up resources for pod-set, but research on determinate genotypes that produce a terminal bud early in the season did not succeed (Patrick and Stoddard, 2010). New strategies to alter plant-level allocation, new perspectives on the phenotype, or both, are needed.

Here, we combined new experiments and published data to achieve two aims. The first was to test the hypothesis that resource availability is the major source of variation in pod-set and yield of faba bean, whereas allocation to pods or between pods is a minor source of variation. Evidence in favour of this hypothesis would support that indeterminate pod-set is not a significant yield constraint *per se*, although it might offer distinct opportunities to control yield-determining processes. The second aim was to clarify how pod-set at the node level is related to the plant- and crop-level phenotype.

## Materials and methods

### Overview

Four studies were designed, each with specific hypotheses that contribute to the main hypothesis in the Introduction. Study 1 combined a multienvironment variety trial and reanalysis of two published datasets to compare the plasticity of seed number and size and test the nature of their trade-off. Study 2 consisted of three new field experiments that test the modularity of nodes during pod-set. Study 3 was a quantitative synthesis of traits derived from the shapes of pod profiles from Study 2 and the literature, testing if variations of the lanceolate pod distribution are associated with yield. Study 4 was a quantitative synthesis of published comparisons of determinate and indeterminate genotypes, aiming to identify whether growth or allocation was the reason for the failure of determinacy to improve yield.

### Study 1: yield and the size–number trade-off of seeds

#### Hypotheses

We hypothesized that (H1) faba bean accommodates variation in environment through seed number more than size; (H2) for a given growth, the seed size–number trade-off has a slope of –1 on a log–log scale; and (H3) seed size and pod mass per seed have a slope of +1 on a log–log scale. Evidence in favour of these hypotheses would imply that selection for pod number is neutral for yield, all else being equal, challenging the notion that pod-set *per se* is relevant to yield improvement.

#### Data sources and statistical analysis

To compare the variation in seed number and size (H1), we used a dataset from the National Variety Trials (NVT), a large database of yield and seed size collected from well-managed, small-plot trials conducted across the Australian cropping regions (http://nvt.grdc.com.au). We identified a set in this database that included nine genotypes completely represented in 55 environments resulting from the partial combination of 18 locations (in South Australia, Victoria, and southern New South Wales) and 6 years (2016–2022). Environmental yield was taken as the average yield of these genotypes and, for each genotype, seed number was derived from measured seed yield and size. Variation in seed number and size was calculated as (maximum–minimum/maximum)×100% (Sadras, 2007; Sadras and Slafer, 2012).

To test the size–number trade-off (H2, H3), we used the method of Henery and Westoby (2001). Data for seed number (seeds m^–2^), seed size (mg per seed), pod wall mass (g m^–2^), and total shoot biomass (g m^–2^) were retrieved from Manson *et al.* (2024a), a dataset consisting of best linear unbiased predictions (BLUPs) predicted from three environments and 21 varieties, the complete historical collection of Australian varieties, and four elite breeding genotypes; experimental and statistical methods are reported in that publication. Seed number was divided by biomass to control for resources, analogously to area of canopy outlined in Henery and Westoby (2001). Seed number per unit of biomass was regressed with seed size on a log–log scale, and the slope of the regression was estimated with reduced major axis regression to account for error in both variables (Niklas, 1994); the slope was considered to deviate from the null hypothesis if –1 did not fall within its confidence interval. Pod wall mass was divided by seed number, then regressed with seed size using the same method, testing if +1 fell within the confidence interval of the slope. We were concerned that the result would not extrapolate to other datasets because flowering time, seed size, and seeds per pod are confounded in this dataset (Manson *et al.*, 2024a), so we applied the same method to data retrieved from Agung and McDonald (1998), an experiment that included a genotype for each combination of early, mid, and late maturity crossed by small, medium, and large seed size. Genotype rankings were very similar for yield between location–years, so we used the means of seed number, size, and shoot biomass of each genotype. Pod wall mass was not reported in this experiment.

### Study 2: modularity of nodes during pod-set

#### Hypotheses

**Fig. 1.**
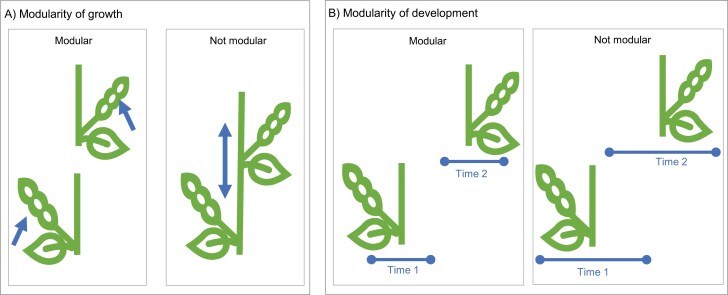
A framework for two sources of modularity for nodes during pod-set. (A) Modularity of growth is where each node produces and consumes its own resources. (B) Modularity of development is where each node starts and completes its development cycle in a unique window. Both sources of modularity could apply, forming two dimensions of phenotypic space. Each source could be a continuous variable on a spectrum, from highly modular to highly non-modular (i.e. pod-set is organized entirely at the node scale to entirely at the plant scale).

A framework for two sources of modularity in pod-set is described in Fig. 1; a justification to use pods per node as a proxy for seed yield per node is provided in Supplementary Table S1. According to this framework, we hypothesized that (H4) nodes are not modular in terms of growth during pod-set, and (H5) nodes are not modular in terms of development during pod-set. We also hypothesize that (H6) manually removed fruits will be replaced higher up the stem and (H7) fruit removal will be neutral or negative for yield. Evidence in favour of these hypotheses would support a model of pod-set in which the hierarchy between pods is the result of a plant-scale system to overproduce and cull fruit according to resource status and current investment in seeds while retaining the ability to replace lost or damaged fruits.

#### Experimental designs

We conducted three experiments in South Australia to complement limited data for the effect of resource augmentation on pod-set in the literature. Experiment I was conducted at Freeling (–34.419, 138.784) in 2022, and Experiments II and III were conducted at Kapunda (–34.409, 138.867) in 2023; these are the same as in Manson *et al.* (2024a) in which data for growing conditions are reported.

Experiment I consisted of a split-plot factorial with a randomized complete block design (RCBD) and three replicates. The main plots were four genotypes: PBA Amberley and PBA Samira, which are adapted to the southern region of Australia with slower phenological development, and PBA Nasma and PBA Nanu, which are adapted to the northern region with faster development. In the subplots, stands were thinned from 22 plants m^–2^ to 4 plants m^–2^ at one of three times: first flowers open, first pods visible (4–5 weeks after flowering), or end of flowering (7–8 weeks after flowering), and a control kept at 22 plants m^–2^ until harvest. Early varieties PBA Nasma and PBA Nanu were thinned a week before late varieties PBA Amberley and PBA Samira.

Experiments II and III were grown adjacent to each other and were identical in design and photothermal regime, but Experiment II was irrigated twice with 30 mm of water, at flowering and 2 weeks after flowering, while Experiment III was rainfed. Based on the results in Experiment I, we removed the genotype factor, using only PBA Samira, a widely adapted variety, and simplified the thinning treatments. Experiments II and III consisted of a split–split-plot factorial with an RCBD and three replicates. The main plots were allocated to stand thinning, from 24 plants m^–2^ to 4 plants m^–2^, or a control maintained at 24 plants m^–2^. The subplots were allocated to time of treatment: 2 weeks after flower emergence or 2 weeks after pod emergence, 4 weeks apart. The sub-subplots were allocated to fruit removal or an undamaged control. Fruit removal involved plucking the racemes from the lowest four nodes on the main stem, including the first flowered node or the first podded node, and we recorded the node number. The treatments were applied to plants that were tagged on the main stem during vegetative growth. The experimental design of Experiments II and III blocked the thinning treatments to minimize edge effects between thinned and not-thinned subplots, and crops were sown by hand on a grid for improved plant spacing.

#### Data and statistical analysis

Measurements were similar across experiments. At early flowering, we measured photosynthetically active radiation (PAR) and the red:far-red ratio immediately above the canopy, at 75% and 50% plant height and at ground level. The measurements within the canopy approximated the region where flowers were open. Three measurements per control plot were taken at each height.

At maturity, we collected five intact plants per subplot (Experiment I) or the four tagged plants per sub-subplot (Experiments II and III). We counted stems per plant and measured pod number, seed number, and individual seed size for all side stems combined. On the main stems we counted pods per node, seeds per node, and measured yield per node. In Experiment I, an extremely high-yielding year at ~10 t ha^–1^, we counted pod number on every main stem node but assessed seed number and yield per node only at nodes 1, 5, 9, and 13. In Experiments II and III, in lower yielding years at ~4 t ha^–1^, we measured all seven pod-bearing nodes. We measured biomass for whole plants and yield for main stems separately from side stems.

To obtain predicted values, we fit linear mixed models with the AsREML-R package (Butler et al., 2023) in the R environment (R Core Team, 2021). Fixed terms were treatments (and node position as a factor, when applicable), random terms were block, main plot, subplot and sub-subplot as needed, and residual terms were experiment row and column. Count data were often skewed, but model assumptions were satisfied after log-transformation. Model predictions were made for significant treatments using the biometryassist package (Nielsen *et al.*, 2023), which provides standard errors and confidence intervals, or approximations of these when data were log-transformed, and conducts a Tukey’s honestly significant difference (HSD) test.

### Study 3: yield and variation in lanceolate pod profiles

#### Traits

**Fig. 2.**
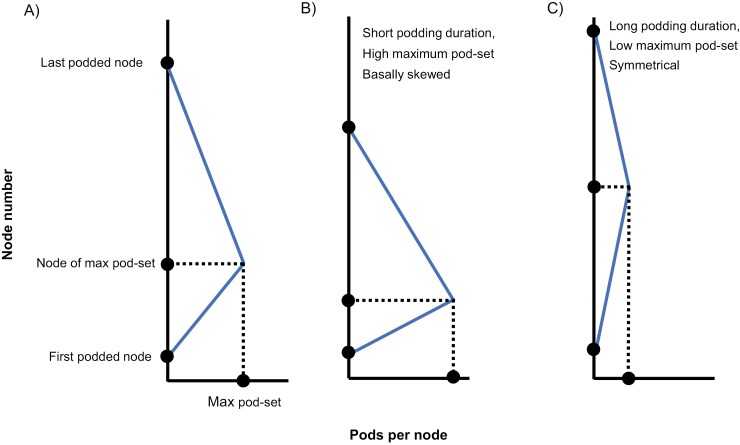
Illustration of pod profile traits retrieved for Study 3. (A) Six traits in lanceolate pod profiles: first podded node, last podded node, podding duration (first minus last podded node), maximum pod-set (pods per node), node of maximum pod-set, and skewness (node of maximum pod-set divided by podding duration). (B) and (C) are paradigmatic illustrations of how variation in the traits relates to variation in the lanceolate distribution.

A ‘pod profile’, or the vertical distribution of pods per node along a stem, has a lanceolate shape in faba bean. We collated a database of pods per node and node number from the literature and Study 2, and extracted the six traits described in Fig. 2. These are inspired by the parameters of a beta function, but we did not fit beta functions because we do not consider curvature a reliable parameter for these data where pods per node is not normally distributed at many nodes (see Supplementary Figs S1–S3), and because we could obtain reliable estimates of traits without fitting a function.

The pod profile traits in Fig. 2B are based on common profiles from spring/late sowing, and those in Fig. 2C are based on common profiles from autumn/early sowing (Stoddard, 1993b); they illustrate how variation in the traits relates to variation in the lanceolate distribution of pods.

#### Hypotheses

We hypothesized that (H8) across environment and sowing date treatments (same genotype and plant population density), yield is associated with podding duration and maximum pod-set but not skewness; (H9) across plant population density treatments (same genotype and environment), crop yield is negatively associated with plant yield and maximum pod-set but not with podding duration or skewness, and (H10) across pollen manipulation treatments (same genotype and environment), pods per main stem is associated with maximum pod-set but not podding duration or skewness. Evidence in favour of these hypotheses supports the notion that yield is a function of pods per m^2^, not pods per plant, and basally skewed pod-set is irrelevant to yield.

#### Data sources and statistical analysis

Crop yield, pod profile data, and agronomic details were retrieved from the sources listed in Supplementary Table S2. Pod profile graphs were digitized using the online app WebPlotDigitizer (https://automeris.io/). We derived the pod-set traits outlined in Fig. 2, and plant yield from crop yield and plant population density. To facilitate comparisons between experiments, we normalized each trait by its average for a given experiment and genotype.

For analysis, we identified three overlapping subsets of treatments in our database: (i) variation in environment (location, year, sowing date, and/or irrigation), for a given genotype and plant population density; (ii variation in plant population density for a given genotype and environment; and (iii) variation in pollen treatments (supplementation or reduction) for a given genotype and environment. For pollen treatments, we used pods per main stem as a proxy for yield that was unavailable for most experiments. We analysed the environment and plant population density subsets separately because the relationships between pod, plant, and population are not comparable, and the pollen subset did not have variation in environment or plant population density.

We tested the direction and significance of the relationships between plant and pod traits with crop yield across experiments in two ways. First, we used least-squares regression on the normalized traits and accepted the outcome if *P*<0.05. Second, as an additional check for collinearity between pod-set variables, we fit a linear mixed model using the AsREML-R package in the R environment. Fixed terms were pod-set traits; the random term was the experiment. We used Wald tests to check the significance of the variables, with *P*<0.05 as the threshold.

We also tested the effects of agronomic interventions on crop yield, plant yield, and pod-set traits. These were sowing date, a subset of the environmental source of variation, and plant population density, the same subset as above. We tested these relationships with least-squares regressions and linear mixed models as above. The pollen treatments were not directly comparable across experiments so we could not test whether pollen supplementation/limitation affected pod number in our database.

### Study 4: synthesis of comparisons of determinate and indeterminate genotypes

#### Hypothesis

We hypothesized that (H11) determinacy failed to increase yield due to an increased allocation to stems at the expense of grain (i.e. a reduction in harvest index). Evidence supporting this hypothesis indicates that other mechanisms are required to improve allocation to pods.

#### Data sources and statistical analysis

Crop yield, shoot biomass at harvest, and stems per plant were retrieved from the literature listed in Supplementary Table S3 and collated in a database. We derived harvest index from yield and biomass. Sources of variation included year, location, sowing date, plant population density, and water regime.

For each indeterminate–determinate pair in a treatment and environment, we plotted the traits and fit reduced major axis regressions to quantify the slope and intercept of their relationship. We also visualized the difference (determinate minus indeterminate for each pair) with frequency distributions. Comparisons of indeterminacy and determinacy in this dataset used varieties with different genetic backgrounds; because determinacy was confounded with other genetic and phenotypic factors, a more complex meta-analysis was not justified.

## Results

### Yield, the variation of seed number and size, and the size–number trade-off (Study 1)

**Fig. 3.**
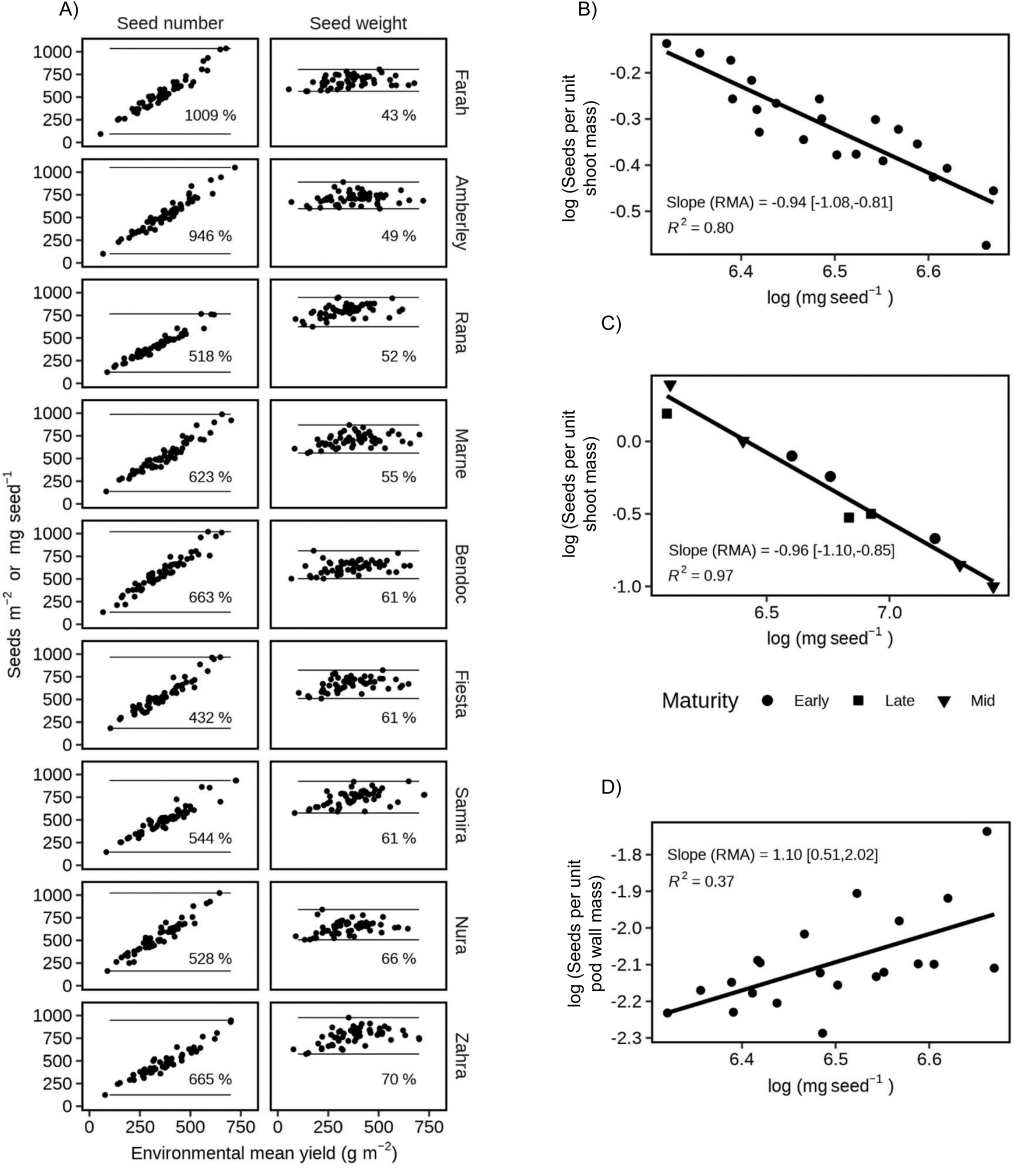
Variation and trade-offs for seed number and size. (A) Association of seed number and size with environmental yield, and their percentage variation, for nine genotypes grown in 55 environments. Data from GRDC National Variety Trials. (B) Trade-off between seed number and size, controlled for shoot biomass (seeds m^–2^ per shoot g m^–2^). Data are from Manson *et al.* (2024a). (C) Trade-off between seed number and size, controlled for shoot biomass (seeds m^–2^ per shoot g m^–2^). Data from Agung and McDonald (1998). (D) Association of accessory cost per seed (seeds m^-2^ per pod wall g m^–2^) and seed size. Data from Manson *et al.* (2024a). RMA, reduced major axis; values in square brackets are the 95% confidence interval.

In the large database of nine genotypes grown under 55 environments, seed number varied from 432% to 1009% and seed size from 43% to 70% (Fig. 3A). The number of seeds per m^2^ increased linearly with favourable environmental conditions, with 0.90≤*R*^2^≤0.94. The regression of seed number and genotype yield also returned 0.90≤*R*^2^≤0.94, and the residual standard error (i.e. the remaining yield variation explained by seed size) had a mean of 34 g m^–2^ for the nine genotypes, compared with a yield range of 50–750 g m^–2^. This supports the notion that faba bean accommodates environmental variation through seed number, with a minor seed size component (H1).

In two independent datasets with different sources of variation, the trade-off between seed number per unit of shoot biomass and seed size had a slope that did not differ from –1 on a log–log scale (Fig. 3B, C), supporting H2. These datasets support the notion that the size–number trade-off for faba bean seeds is neutral for yield.

The association of seed number per unit pod wall mass and seed size had a slope that did not differ from +1 (Fig. 3D, H3). This further supports the notion that the size–number trade-off for faba bean seeds is neutral for yield, adding the insight that, for these genotypes and environments, there was no effect on yield by how seeds were packaged into pods.

### Yield response to canopy thinning and fruit removal (Study 2)

In rainfed crops in 2023 (Experiment III), fruit removal was neutral or negative for yield. In the irrigated crops of 2023 (Experiment II), fruit removal when the first four racemes had open flowers caused the plants to overcompensate yield, increasing from 27.8 g per plant in the control to 41.3 g per plant (Table 1). This yield overcompensation of 49% was associated with greater biomass (41%), seed number (32%), and pod number (28%), and a slight increase in harvest index (8%). Overyielding was associated with side rather than main stem yield. Plants did not compensate for fruit removal at podding (Table 1).

**Table 1.** Response of plant yield and components to fruit removal at two timings (Experiment II)

			Predicted mean (SE)	
Level	Trait (unit)	Timing	Control	Fruit removed
Plant	Yield (g per plant)	Flowering	27.8 (1.2) b	41.3 (1.3) a
		Podding	24.9 (1.2) b	17.8 (1.2) c
	Biomass (g per plant)	Flowering	55.2 (3.1) b	77.6 (3.3) a
		Podding	51.2 (3.0) b	47.0 (3.1) b
	Harvest index (fraction)	Flowering	0.48 (0.01) ab	0.52 (0.01) a
		Podding	0.48 (0.01) b	0.38 (0.01) c
	Seed number (seeds per plant)	Flowering	42.8 (2.5) b	58.6 (2.5) a
		Podding	35.24 (2.6) bc	30.80 (2.5) c
	Seed size (mg per seed)	Flowering	669.3 (25.7)	679.6 (25.6)
		Podding	690.7 (25.1)	639.6 (26.7)
	Pod number (pods per plant)	Flowering	17.7 (1.0) b	22.7 (1.0) a
		Podding	15.3 (1.0) bc	12.62 (1.0) c
	Stem number (stems per plant^-1^)	Flowering	2.8 (0.2)	3.2 (0.2)
		Podding	3.2 (0.2)	2.9 (0.2)
Main stem	Yield (g stem)	Flowering	8.1 (0.8) a	12.4 (1.3) a
		Podding	9.8 (1.0) a	3.1 (0.3) b
	Pod number (pods per stem)	Flowering	6.8 (0.5) a	6.6 (0.5) a
		Podding	5.9 (0.5) a	2.1 (0.5) b
Side stem	Yield (all stems) (g per plant)	Flowering	15.1 (1.2) b	26.4 (2.0) a
		Podding	14.2 (1.1) b	15.4 (1.2) b
	Yield (per stem) (g per stem)	Flowering	9.1 (0.6)	12.3 (0.8)
		Podding	7.1 (0.5)	8.8 (0.6)
	Pod number (pods per stem)	Flowering	6.4 (0.4)	7.3 (0.4)
		Podding	4.6 (0.4)	5.3 (0.4)

Predicted means combine control and thinned stands because the three-way interaction was not significant for most traits. Numbers in parentheses are the SE of the predicted mean, and letters distinguish groups by Tukey’s HSD for removal:timing treatments; absence of letters indicates no differences. Green shading indicates a positive response in a trait to fruit removal, and orange shading indicates a negative response.

The results support H6 with qualification: manually removed fruits can be restored given adequate time and resources. Against H7, overyielding observations indicate that it is possible for fruit removal to increase yield in some growing conditions.

### Pods per node response to canopy thinning and fruit removal (Study 2)

**Fig. 4.**
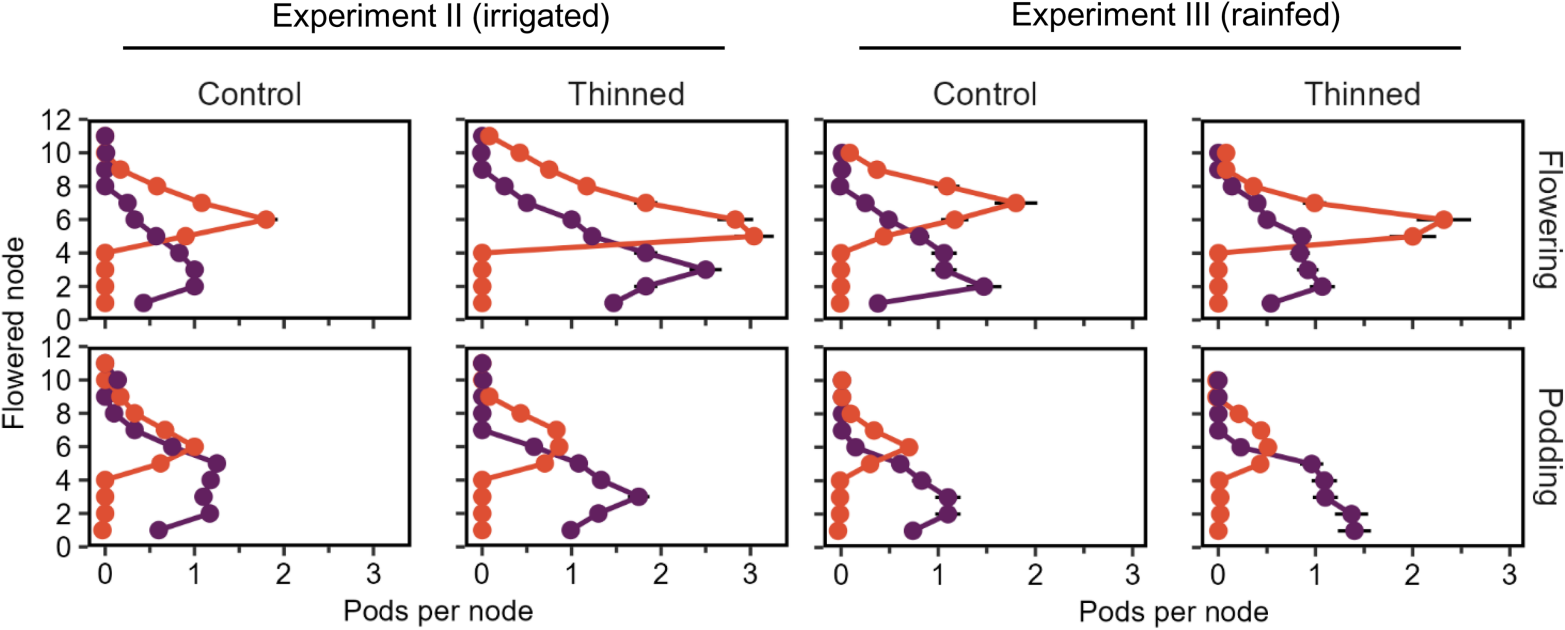
Main stem profiles of mature pods in untreated plants (purple) or plants with racemes removed (red) at one of two stages, flowering (top row) or podding (bottom row), in intact stands (control, 24 plants m^–2^) and stands thinned to 4 plants m^–2^ (thinned) in two experiments. In the ‘Flowering’ treatment, the first four racemes with open flowers were removed and in the ‘Podding’ treatment (~4 weeks after flowering), the first four racemes with visible pods were removed. Stand thinning was applied at the same timings. Error bars are 2 SEs of the prediction. Nodes were counted from the first flowered node of each plant. Data from Study 2, Experiments II and III in 2023.

In each raceme removal treatment of Experiments II and III, fruits were replaced at higher nodes (Fig. 4), consistent with H6. Maximum pod-set was increased by higher resource availability (Supplementary Table S4).

**Fig. 5.**
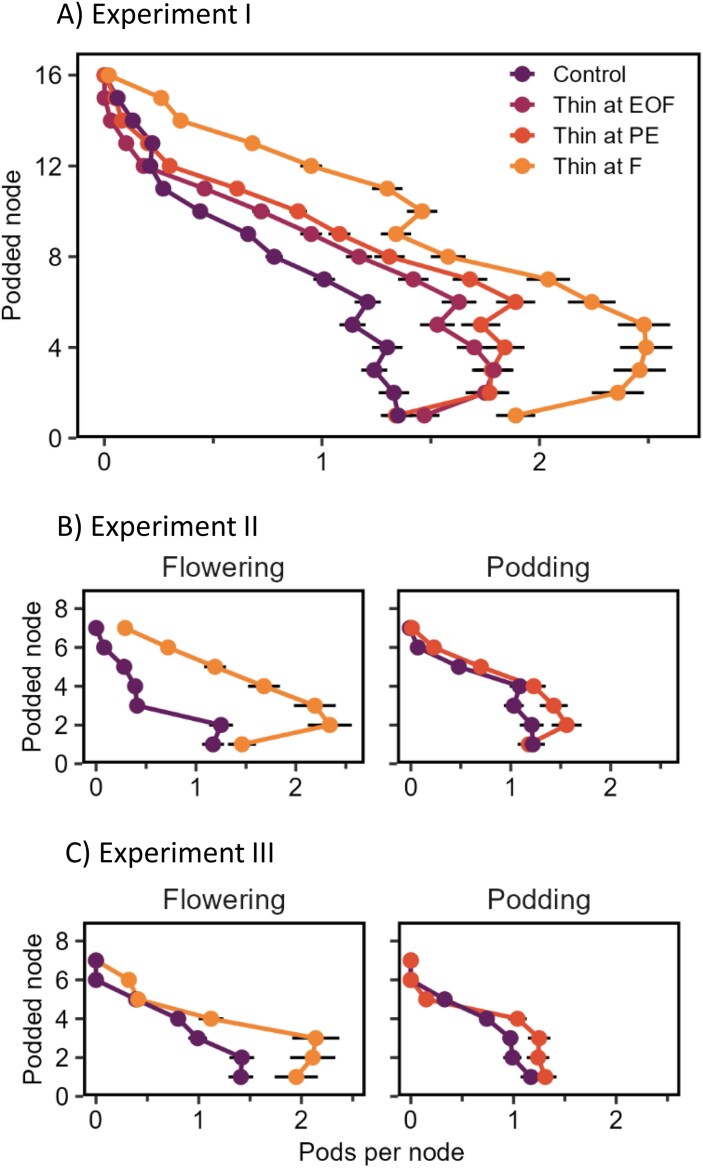
Main stem profiles of mature pods in response to stand thinning (from ~23 plants m^–2^ to 4 plants m^–2^) in Experiments I (A), II (B), and III (C). In (A), timing of thinning is flowering (F), pod emergence (PE), or end of flowering (EOF). In (B) and (C), predictions are for the undamaged control plants only. Error bars are 2 SEs of the prediction. Nodes were counted from the first podded node of each plant. Data from Study 2.

Pods per node responded to stand thinning at most nodes of the main stem in Experiments I, II, and III, as did maximum pod-set (Fig. 5; Supplementary Table S5). Remarkably, pod number on the lowest nodes responded to stand thinning even though treatments were applied 4–8 weeks after these flowers had opened. This strongly supports a lack of node modularity in terms of development during podding (H5, Fig. 2B). Podding duration varied by only one or two nodes in response to stand thinning and, although skewness ranged from 0.06 to 0.50, it had no association with plant yield.

### Extinction of radiation and the red:far-red ratio in the canopy (Study 2)

**Fig. 6.**
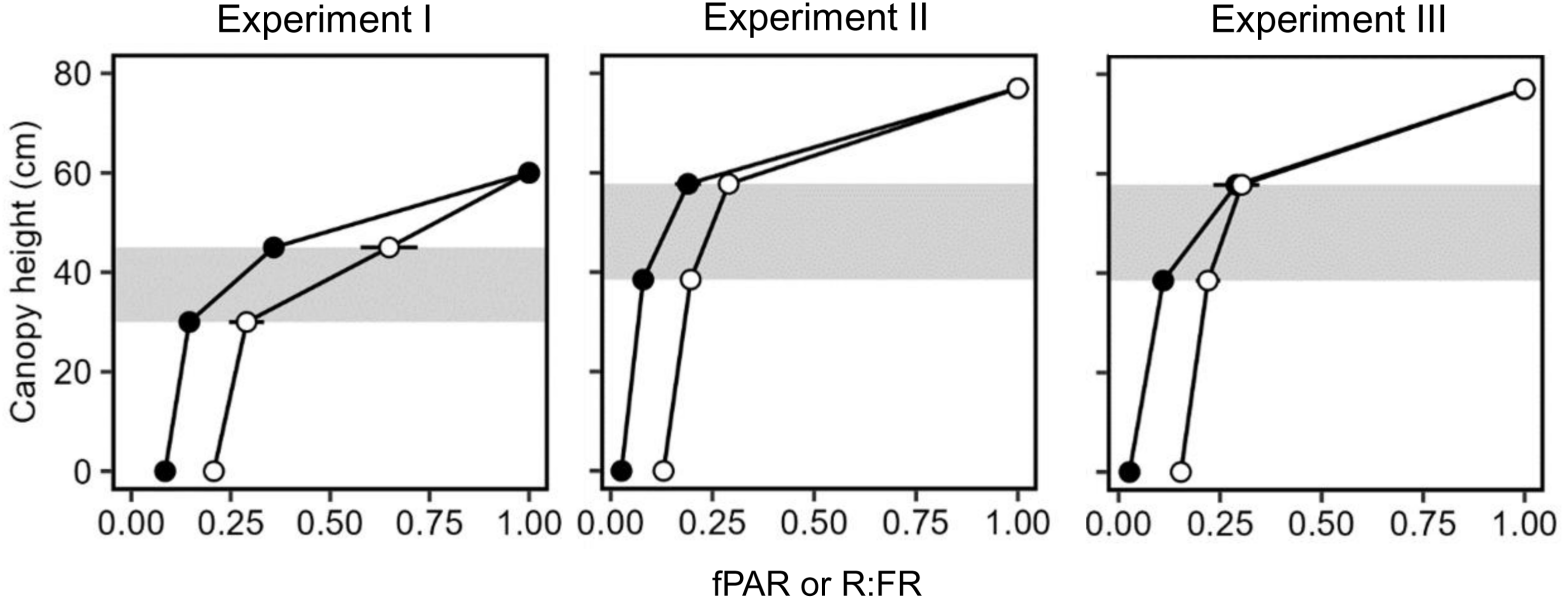
Profiles of radiation extinction (fPAR, fraction of photosynthetically active radiation, filled circles) and red:far-red ratio (R:FR, open circles) measured at flowering in canopies of control treatments from Experiments I, II, and III. Each point is the mean of three replicates; error bars are 2 SEs. The grey area is the approximate region of the canopy with open flowers.

The extinction of PAR and R:FR in intact faba bean canopies is shown in Fig. 6. At flowering, the section of the profile where flowers were open received 10–30% of maximum PAR, and R:FR in this layer was 0.2–0.6. With PAR ~100–300 µmol m^–2^ s^–1^, photosynthetic rate would be ~25% of maximum net photosynthetic rate in the region in which pod-set is greatest (Avola *et al.*, 2008), strongly supporting the lack of node modularity in terms of growth (H4, Fig. 2A).

### Associations of pod distribution with plant and crop yield (Study 3)

**Fig. 7.**
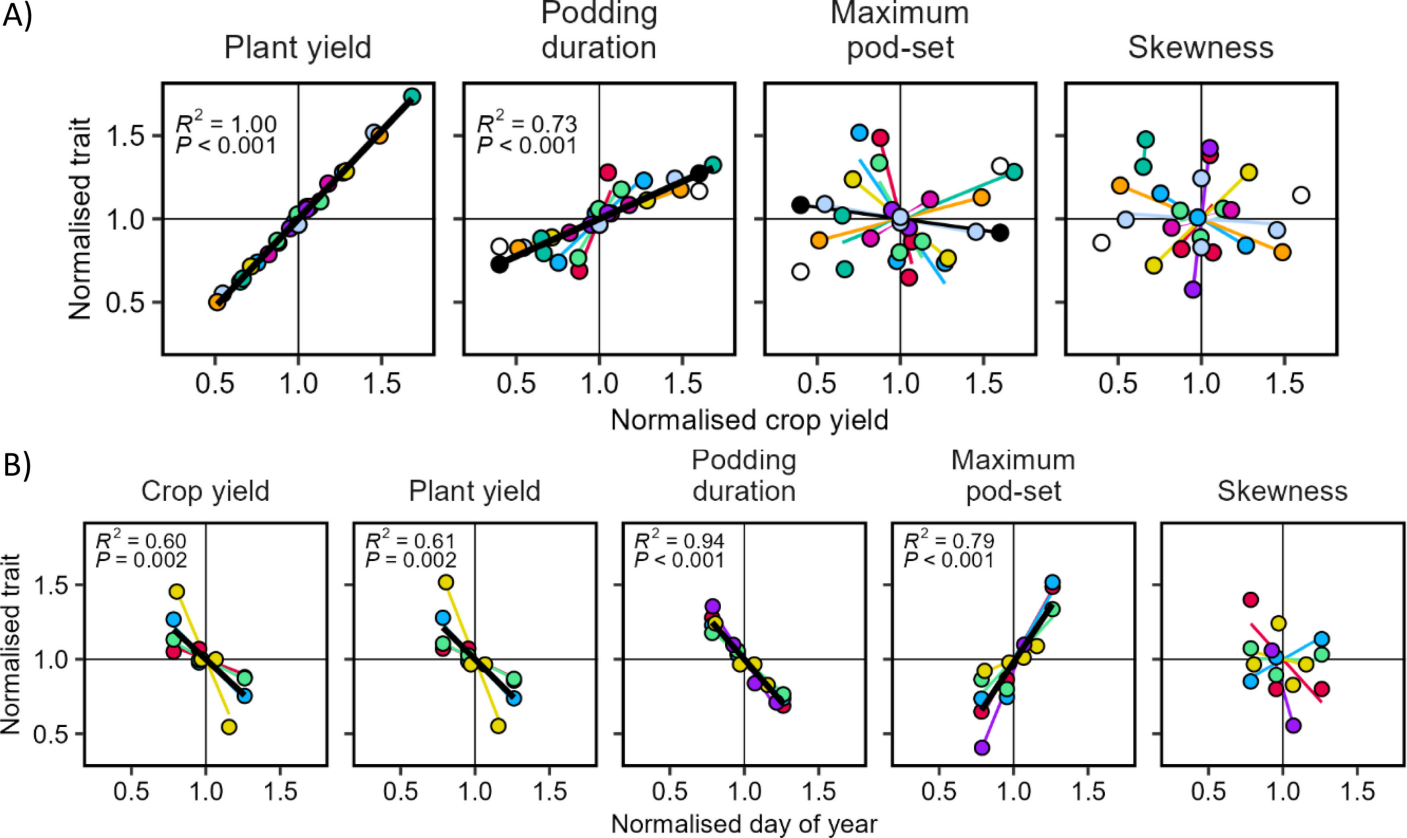
Pod profile traits for environmental sources of variation. (A) Association with crop yield. (B) Association with sowing date. Colours are individual experiments, lines are least-squares regressions, and a black line is drawn across experiments when *P*<0.05 for the whole dataset. Podding duration is the number of nodes from first to last podded node, maximum pod-set is the largest pod number of the profile, and skewness is the node where maximum pod-set occurred divided by the number of podded nodes (i.e. 0.1 is skewed to the bottom, 0.5 is symmetrical, 0.9 is skewed to the top). All traits are normalized by the average of the experiment. Data from Study 3.

For environmental sources of variation associated with location, season, sowing date, and water regime, crop yield was correlated with plant yield and podding duration, but not maximum pod-set or skewness (Fig. 7A). In a linear mixed model of the pod profile traits, only podding duration was associated with crop yield (*P*<0.001). For the sowing date subset of our database, later sowing consistently reduced podding duration, which was compensated for by a higher maximum pod-set with no association for skewness (Fig. 7B). The net result of the changes to the pod profile was a reduction in crop and plant yield with later sowing. In the linear mixed model, the significant terms were also maximum pod-set (*P*=0.017) and podding duration (*P*=0.008). The results support H8 that predicted an association of podding duration and maximum pod-set with environment.

**Fig. 8.**
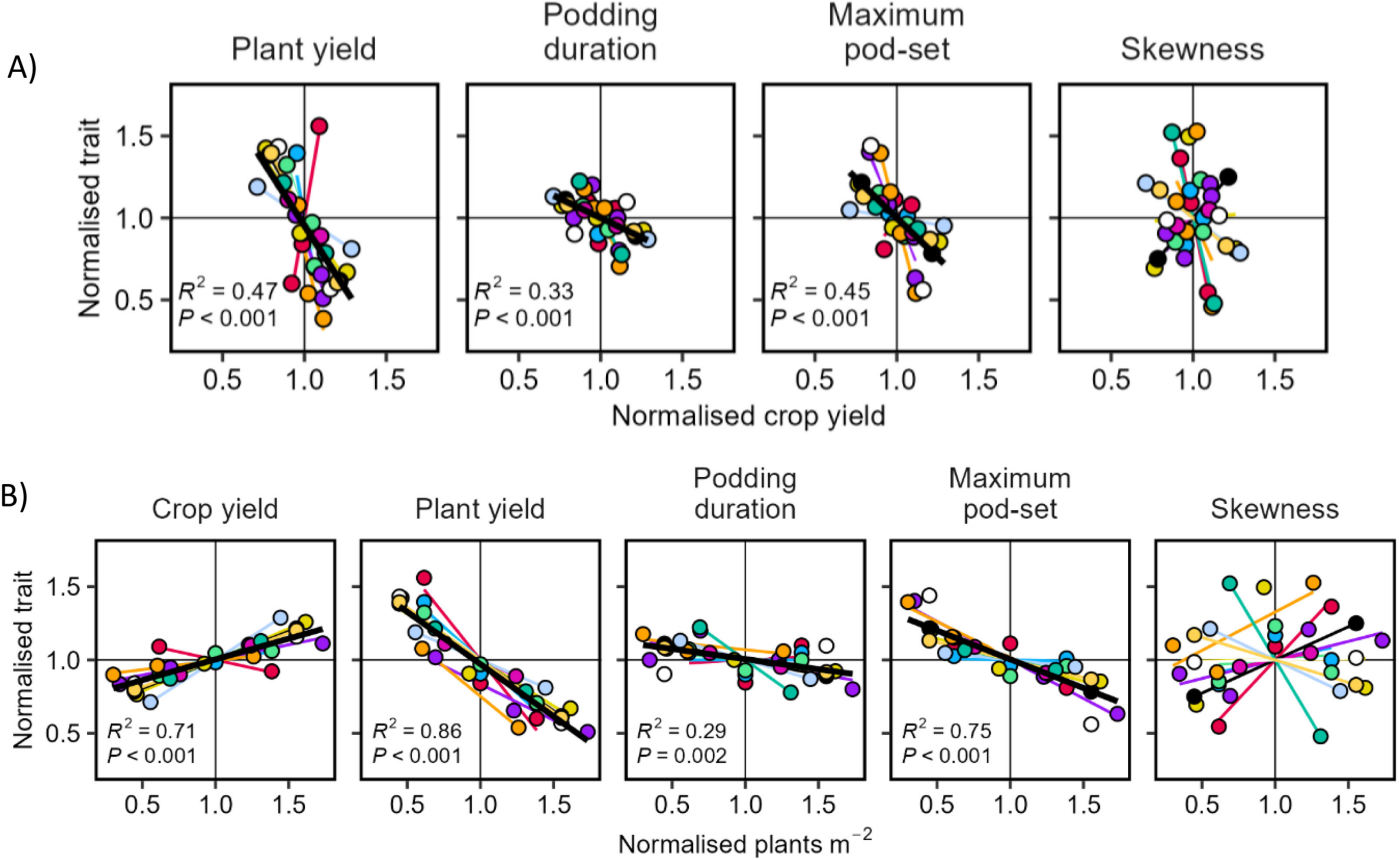
Pod profile traits for plant population density as the source of variation. (A) Association with crop yield. (B) Association with plant population density. Podding duration is the number of nodes from first to last podded node, maximum pod-set is the largest pod number of the profile, and skewness is the node where maximum pod-set occurred divided by number of podded nodes (i.e. 0.1 is skewed to the bottom, 0.5 is symmetrical, 0.9 is skewed to the top). All traits are normalized by the average of the experiment. Colours are experiments; a black line is drawn across experiments when *P*<0.05. All lines are least-squares regressions. Data from Study 3.

Increased plant population density increased crop yield at the expense of plant yield and maximum pod-set (Fig. 8). To a lesser extent, there was also a reduction in podding duration. In the linear mixed model of pod profile traits, significant terms were maximum pod-set (*P*<0.001) and podding duration (*P*=0.011). The results support our prediction in H9 for plant yield and maximum pod-set, but we did not anticipate a reduction in podding duration.

**Fig. 9.**
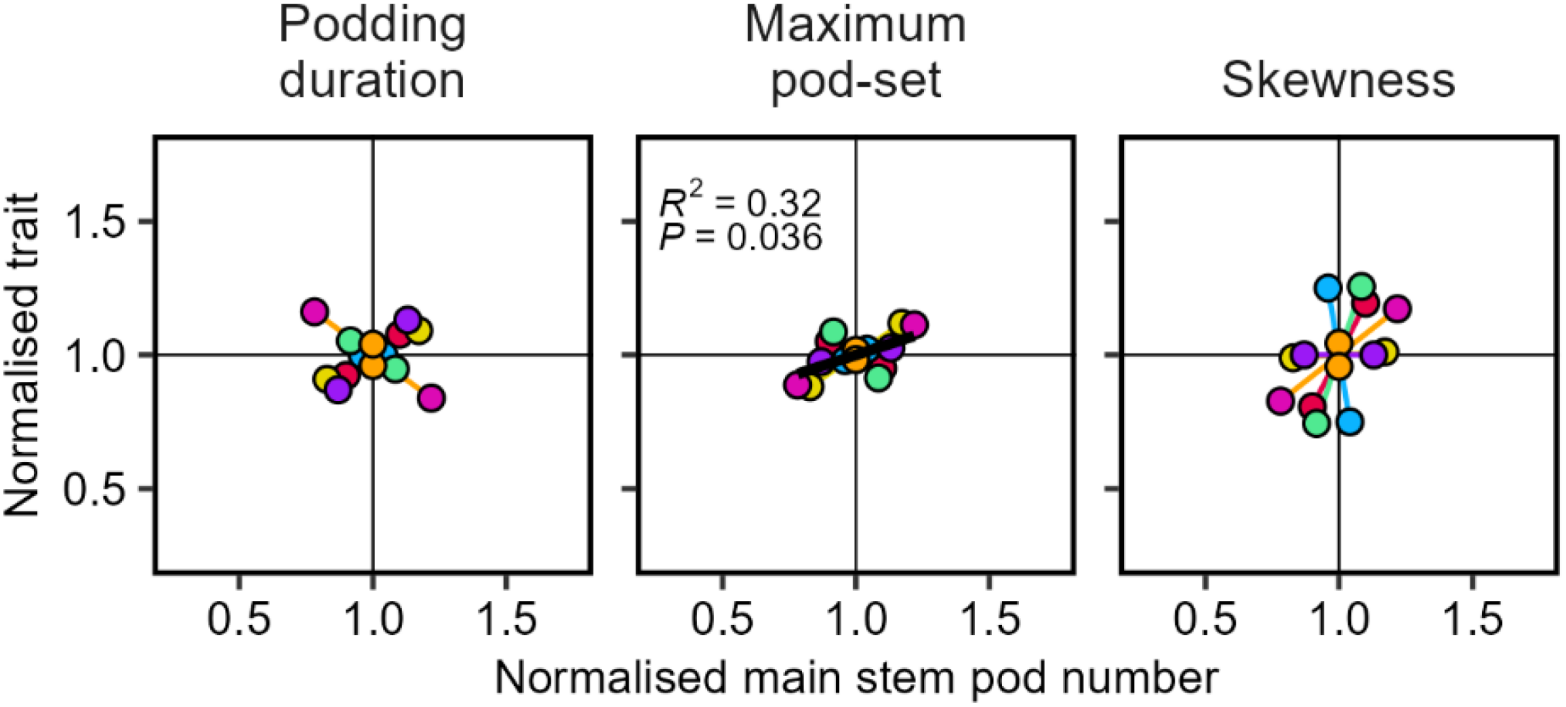
Association of pod profile traits with main stem pod number (a proxy for yield), for pollen manipulation as the source of variation. Podding duration is the number of nodes from first to last podded node, maximum pod-set is the largest pod number of the profile, and skewness is the node where maximum pod-set occurred divided by number of podded nodes (i.e. 0.1 is skewed to the bottom, 0.5 is symmetrical, 0.9 is skewed to the top). All traits are normalized by the average of the experiment. Colours are experiments; a black line is drawn across experiments when *P*<0.05. All lines are least-squares regressions. Data from Study 3.

When pollen supplementation or limitation was the source of variation, only maximum pod-set was associated with main stem pod number, supporting H10 (Fig. 9).

A comparison of Figs 7 and 8 shows that plant and crop yield are coupled across environments, whereas they are negatively related across densities. Thus, the consistent factor for both sources of variation is that pods per m^2^ is associated with crop yield per unit area, the target trait in modern agriculture.

In summary, our results across sources of variation support that yield is a function of pods per m^2^, not pods per plant, and allocation between pods along a stem (i.e. putative competition) is not relevant to yield.

### Synthesis of comparisons of determinate and indeterminate genotypes (Study 4)

**Fig. 10.**
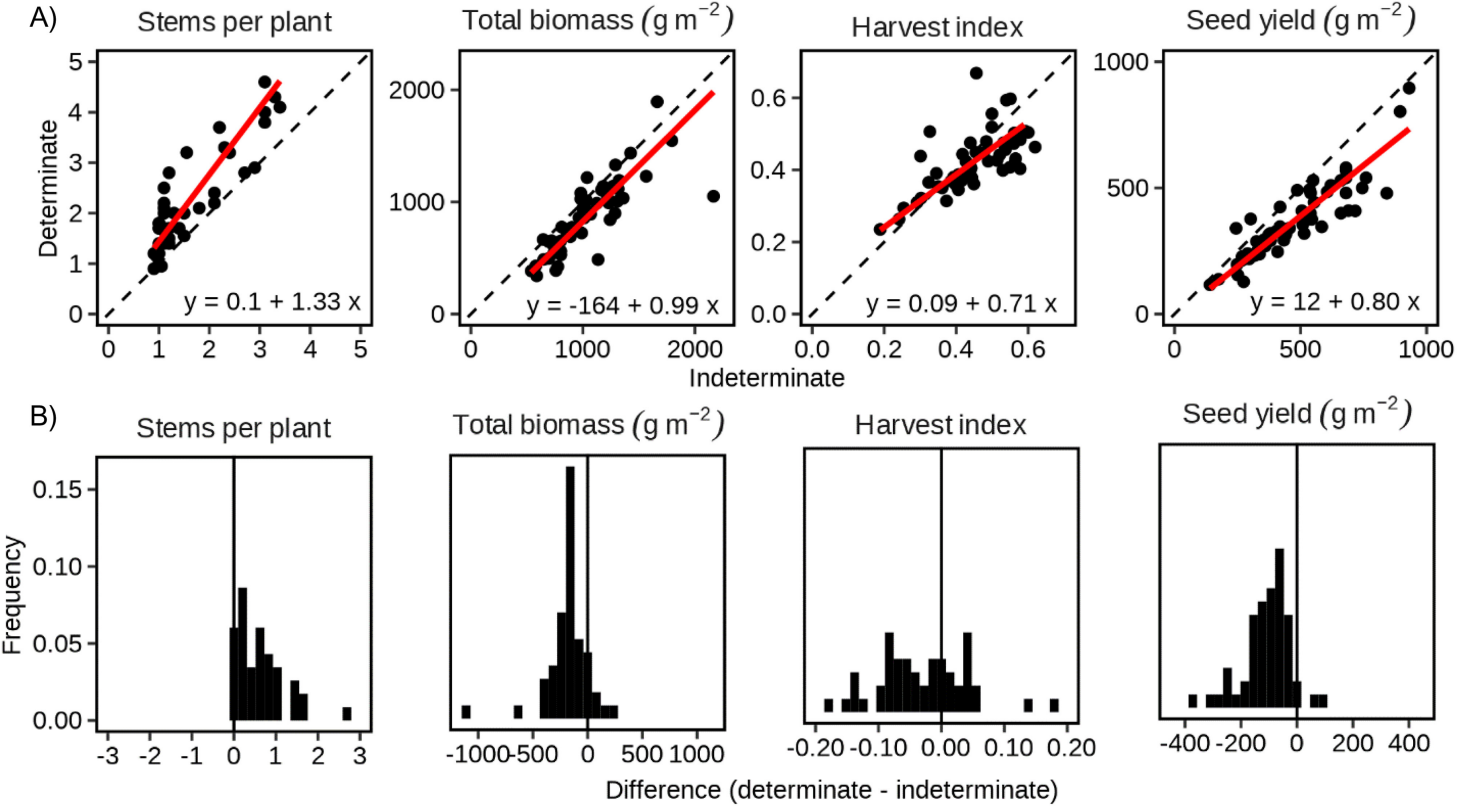
Pairwise comparisons of indeterminate and determinate faba bean varieties across environmental and management sources of variation. (A) Pairwise trait comparisons. Lines are reduced major axis (RMA) regressions, with the equation given in the bottom-right corner and confidence intervals of parameters in Supplementary Table S6. (B) Frequency distribution of pairwise trait differences. Data from Study 4.

For pairwise comparisons of determinates and indeterminates in 43–56 unique combinations of environment and management, determinate varieties produced more stems per plant, less biomass at harvest, and lower seed yield than indeterminate varieties (Fig. 10). The harvest index of determinates was lower in 66% of comparisons but was slightly higher in contexts with low harvest index. We conclude that determinates have lower yield than indeterminates because their greater stem number was consistently associated with lower growth, not predicted in H11, and lower harvest index in most comparisons, in partial support of H11.

## Discussion

### Pod-set is driven by growth

We showed that faba bean yield responds to environmental variation through seed number much more than seed size (Fig. 3A); pod-set is related to yield through the close association of pod number with seed number. Our four studies support our main hypothesis that pod-set and yield are driven by resource availability and crop-level growth. In Study 1, we found in two independent sources of data that dividing growth between seed number, seed size, and pod wall mass was neutral for yield, with genotypes varying in phenology, seed size, and seeds per pod (Fig. 3B–D). In Study 2, fruit removal increased growth to result in overcompensation of pod number and yield (Table 1). In Study 3, favourable environments increased plant podding duration and crop yield (Fig. 7), but plant density increased crop yield despite the reduction in plant yield and pod-set (Fig. 8). In Study 4, determinacy compromised growth and yield (Fig. 10).

Our results are consistent with previous research. Shoot biomass is strongly associated with yield in a wide range of environments globally (Sinclair *et al.*, 2022; White *et al.*, 2022; Manson *et al.*, 2024a) and, while harvest index of faba bean varies (Unkovich *et al.*, 2010), the associations of biomass with yield, seed number, and pod number are stronger (Lake *et al.*, 2019; White *et al.*, 2022; Manson *et al.*, 2024a). We did not find reports of flower mass in the literature, but we estimate that flower mass is <5% of total shoot biomass based on removed racemes of Experiments II and III, Study 2, so flower overproduction and a low flower:pod ratio of 0.06–0.60 (Alharbi and Adhikari, 2020) represents a small cost to faba bean. A relationship between yield and how seeds are packaged across pods has been discussed (Agung and McDonald, 1998) but is not widely documented and would benefit from explicit tests against the null hypothesis of a slope of –1 on a log–log scale (Henery and Westoby, 2001).

In light of this evidence, flower abortion of faba bean might not be a problem to be solved, but is better understood as the nearly universal behaviour of plants to overproduce flowers and serially abort them to a final seed number according to resource status and current investment, retaining the option to replace lost or damaged fruits (Lloyd, 1980; Stephenson, 1981; Sadka *et al.*, 2023). Future research in faba bean should begin with a model of pod-set explained by growth, focusing on growth around early podding, the critical period (Lake *et al.*, 2019). Deviation from this model indicates a minor determinant of yield and an opportunity to improve yield apart from increased growth (Sadras and Dreccer, 2015). Examples of deviations include: genotypes that allocate more critical period growth to seeds than pod walls (Sadras *et al.*, 2019), heat or frost stress that causes flower sterility in addition to its reduction of growth (Farooq *et al.*, 2017), or pollen limitation for pollinator-dependent genotypes with abundant resources (Stoddard, 1993a).

### Podding nodes are not modular for pod-set

Our work is the first to directly address the question of modularity between podding nodes of faba bean. Previous studies of pod interactions assumed a high degree of interaction between pods (Aufhammer and Götz-Lee, 1991), and studies linking pod-set to environment assumed a high degree of modularity, with pod-set linked to short-term weather conditions early in the development of each node (Stoddard, 1993a; Manning *et al.*, 2024), or local radiation interception at each node (Ishag, 1973).

We showed that faba bean nodes are not modular in terms of development, because early nodes responded to late resource augmentation (Fig. 5). Additional, indirect evidence that development is not modular is that the pod profile is always lanceolate and therefore not simply a function of transient weather (Supplementary Table S2). We also showed that nodes are not modular in terms of growth, because most pod-set occurs deeper in the canopy where local light and photosynthesis are not sufficient for pod growth (Fig. 6) (Hodgson and Blackman, 1957; Avola *et al.*, 2008). In further support that growth is not modular, it is well documented that, in dense canopies (Crompton *et al.*, 1984), carbon fixation and leaf nitrogen content are initially greatest in the upper part of the canopy where light intensity is highest, then assimilates are redistributed to pods during seed fill (Austin *et al.*, 1981; Crompton *et al.*, 1981; Dekhuijzen and Verkerke, 1984; Del Pozo and Dennett, 1999).

On the other hand, nodes are modular in terms of fertilization because pollen cannot be redistributed once deposited in a flower. Autofertile genotypes tend to have pod profiles that are more basally skewed than those of cross-pollinating genotypes (Stoddard, 1986; Bishop *et al.*, 2020), seemingly supporting that node-level fertilization determines the phenotype. However, it is confounded with heritable variation in skewness among autofertile genotypes, supporting a whole-plant perspective (Adcock and Lawes, 1976). Furthermore, modularity of fertilization did not drive the phenotype in our data (Fig. 9). We conclude that lack of modularity for growth and development over-rides modularity of fertilization.

If pod-set cannot be investigated at the node-level only, there are three important implications for faba bean research. First, augmenting light penetration into the canopy may not be advisable; assimilates are distributed widely across a plant, there is limited scope to change the shape or orientation of legume leaves (Mandl and Buss, 1981), and reduced plant population density risks compromising light interception and crop yield (Manson *et al.*, 2024b). Second, it is incorrect to link phytomer-level pod-set to short-term weather conditions alone; the effect of external conditions on the probability of pod-set at a phytomer is nested within the effect of existing fruit load and storage capacity of the stem (Ismail and Sagar, 1981). Third, matching faba bean development to environment should focus on maximizing growth and avoiding stress for the critical period as a whole. Node-level interactions may become relevant in the context of recovery after stress.

### Fruit–mother feedback modulates resource capture

In irrigated Experiment II, we observed overyielding in response to fruit removal, associated with replaced pods on main stems, greater pod-set on side stems, and increased biomass. Overyielding after fruit removal was reported in independent experiments, where it was also contingent on adequate resources per plant (Aufhammer *et al.*, 1987; Aufhammer and Götz-Lee, 1989, 1991); time available for recovery is also important.

In species with a racemous or cymous inflorescence type, the infloretic meristems that do not convert to a floral meristem eventually enter ‘global proliferative arrest’, ending phytomeric growth and beginning senescence (Hensel *et al.*, 1994). In this state, meristems remain structurally unchanged but cease cell proliferation (Hensel *et al.*, 1994). It has been known at least since 1928 that fruit development modulates the timing of global proliferative arrest and senescence (Leopold *et al.*, 1959). In cotton, the negative feedback from bolls to shoot apices is known as ‘cut-out’ and is managed to match crop maturity to the prevailing environment (Roche *et al.*, 2024). The phenotype is also observed in soybean, for which photoperiod and photoperiod sensitivity affected yield through postponed pod-set, increased node number, prolonged reproduction, and increased resource capture (Nico *et al.*, 2015, 2016, 2019).

It appears that our fruit removal treatments modulated a similar feedback system in faba bean. In our experiments, we could not count node number reliably in our brittle harvest samples, so we infer that experimental removal of racemes released negative feedback from fruits to shoot tips and was expressed as greater node number, growth, and yield, given adequate resources and time. An alternative, complementary explanation for overyielding is that augmented pod-set on side stems stimulated a higher photosynthetic rate (Yan *et al.*, 2013). In either case, overyielding after fruit removal shows that the relationship of pod-set and resource capture can be represented by a double-headed cause-and-effect arrow, albeit with a larger effect of resource capture on pod-set than in the other direction.

Recent research is clarifying the hormonal, genetic, and environmental mechanisms that control the end of flowering and senescence in Arabidopsis, canola, and field pea (Wuest *et al.*, 2016; Balanzà *et al.*, 2019, 2023; Ware *et al.*, 2020; González-Suárez *et al.*, 2023; Walker *et al.*, 2023; Burillo *et al.*, 2024). There may be an exciting opportunity to manipulate the fruit–mother feedback system of faba bean to increase yield for target environments, perhaps through photoperiod sensitivity as has been suggested for soybean (Nico *et al.* 2019).

### Fruit–fruit feedback is only relevant to crop yield through stress recovery

The feedback from fruit to mother is distinct from that among fruits; it is plausible that dominance from older to younger pods limits yield, but our evidence does not support this for three reasons. First, there was no association of pod distribution and yield when resource or pollen availability was the source of variation (Figs 7–9). Second, the ability to replace lost pods was limited by resources and time (Fig. 4). Third, our instance of overyielding after fruit removal was surprisingly associated with increased biomass; that is, a fruit–mother effect. The evidence is more compatible with the view that suppression of younger pods is yield-neutral; plants allocate limited resources to the most promising offspring, holding younger siblings in reserve to grow only if their elders are lost.

Our view that dominance among pods is yield-neutral, apart from stress recovery, differs from much of the literature for indeterminate crop species, and there are three points that should be clarified in future experimental research. First, some have suggested that competition within a node can be bypassed by increasing podded node number (Nico *et al.*, 2019); given our findings for faba bean, there is an assumption of modularity here that needs closer scrutiny. Second, some have suggested that synchronous pod-set can mitigate competition to increase yield, but it will not do so unless it stimulates a measurable increase in resource capture or allocation to yield. In maize, treatments that increased the synchronicity of pollination increased kernel set but not growth or allocation, so kernel weight decreased and yield was unchanged (Uribelarrea *et al.*, 2008). Third, we argue that a mass-based measurement of allocation such as yield per unit of shoot growth, or yield per unit of critical period growth, is necessary to prove that fruit–fruit feedback has affected yield. Otherwise, yield increases accompanied by increased biomass favour our interpretation that maternal growth drives pod-set and yield, and that fruit–fruit feedback is agronomically irrelevant except after a stress event. In Box 1, we expand the discussion with emphasis on the unjustified notion of ‘competition’ to describe interactions between growing pods.

Box 1. **Lanceolate profiles: the result of competing parts, self-organization, or a coordinating whole?**

A lanceolate distribution of fruit or seed on a mature stem, associated with developmental age, is strikingly common in plants, including faba bean, field pea (Jeuffroy and Chabanet, 1994), soybean (Ceccon *et al.*, 1994), wheat (Tamagno *et al.*, 2024), cotton (Sharma *et al.*, 2015), and grapevine (Haj-Yahya *et al.*, 2024). Here, we outline the mechanisms that contribute to a hierarchy between fruit positions and critically assess the use of the term ‘competition’ to describe those mechanisms.

Dominance partially arises from the activity of seeds. In the past, it was debated if seeds compete for resources directly or if this is mediated by hormonal signalling (Stephenson, 1981; Bangerth, 1989). New models are holistic, showing how carbon balance, nutrient status, and hormonal signalling affect the capacity of a sink to establish its access to resources in the context of other developing sinks; similar processes seem to be involved for shoots and seeds (Barbier *et al.*, 2019; Aloni, 2021; Sadka *et al.*, 2023). These insights provide a useful clarification: at early developmental stages, when variation in sink demand is small and resources are evenly distributed across all candidate sinks, dominance can still be established hormonally, usually according to age; at later stages when size discrepancies are larger, resource pre-emption through unequal sink strength may become apparent as well.

However, this may exaggerate the independence of seeds because the lanceolate profile is also shaped by the mother. Various plant hormones involved in fruit-set or abortion are derived from maternal tissues (Ravishankar *et al.*, 1995). Wheat up-regulates genes associated with programmed cell death of its offspring in response to long days (Ghiglione *et al.*, 2008). Abscission of seeds involves the genetic expression of the mother (Crick *et al.*, 2022). The developmental plan of the mother staggers the initiation of seed growth, providing the initial conditions for size differences to propagate between seeds (Backhaus *et al.*, 2023). This can increase maternal fitness by controlling sibling rivalry (Ganeshaiah and Shaanker, 1988), analogously to staggered clutches in animals (Mock and Parker, 1997), although maternal control can break down at senescence (Dogra and Dani, 2019).

A further issue with the term ‘competition’ is that it implies that the evolutionary interests of the parts are not aligned. Stems and leaves are genetically identical and have the same fitness goals, so competition is certainly the wrong term, and it is only partially applicable to seeds that partially differ in genetic composition from their mother and siblings (at least in outcrossing species such as faba bean). In this case, the concepts of mother–offspring conflict, father–mother conflict, and sibling rivalry may apply (Sadras and Denison, 2009), and it follows that pod or seed abortion could be associated with infanticide (Sakai, 2007), siblicide (Walker *et al.*, 2021), or both (Cailleau *et al.*, 2018). A key test to justify the term ‘competition’ in this sense is to create or identify variation in genetic relatedness of seeds within a plant and see if it is associated with pod or seed abortion.

Shifting emphasis from seeds to plant could still overemphasize agency altogether. Temporal patterns in the environment are correlated with node position (Haj-Yahya *et al.*, 2024). Distal fruit positions tend to hold fewer ovules than proximal positions from the beginning, a passive ‘architectural effect’ (Diggle, 1995; Jadeja and Tenhumberg, 2018). Lanceolate profiles could result from ‘self-organization’—an emergent property of developmental rules for seed growth that propagate random initial size differences to the phenotype at maturity (Ganeshaiah and Shaanker, 1992).

In summary, the assumption of competition could misassign agency to plant parts over the whole or overemphasize agency altogether and lead to inefficient research agendas. Instead, agency from seeds, agency from mother plants, and passive processes are plausible hypotheses that should be explicitly tested or tentatively invoked to explain lanceolate profiles.

### Inflorescence architecture for pod-set

Determinacy was pursued in faba bean to reduce putative competition between shoot apex and pods and to increase yield stability, which was achieved at the expense of average yield (Chapman, 1981; De Costa *et al.*, 1997). Our synthesis (Fig. 10) showed that determinate faba bean plants consistently produce new stems that partially compensate for fewer nodes per stem; the compensation is partial with a net reduction in growth and yield because these extra stems emerge late in the season with reduced time and space to grow (Nagl, 1981). Instead, the evidence supports that resource availability and growth are more important to pod-set than putative competition between pods and shoots. Across environments, yield was associated with podding duration (Fig. 7), and the yield advantage of indeterminates increased with environmental yield (Fig. 10). We found that fruit removal benefited yield by limiting fruit–mother feedback, the opposite of determinacy to limit mother–fruit feedback. In de-topping experiments, pod-set on remaining nodes increased but yield decreased (Hodgson and Blackman, 1957; Chapman *et al.*, 1978; Saxena *et al.*, 1981).

Perhaps semi-determinacy, namely the formation of a terminal bud at a node midway between determinate and indeterminate faba bean (Stoddard, 1993b), would strike a balance between yield plasticity and allocation to seeds. Semi-determinacy has shown promise in soybean (Bernard, 1972), chickpea (Ambika *et al.*, 2021), and tomato (Vicente *et al.*, 2015). Australian varieties have been called semi-determinate because they are reported to cease phytomeric growth before genotypes from other backgrounds, but this is poorly documented (GRDC, 2017). It may also be incorrect because they do not produce a terminal bud; perhaps they reflect variation in the sensitivity of shoot tips to pod-set and would better be characterized as ‘semi-indeterminate’. Semi-dwarfism might retain plasticity in node number but reduce allocation to stems (Hughes *et al.*, 2020).

### Management for pod-set

Crop yield is a population trait, and our results support that management should aim to maximize crop-level resource availability and growth. Where this is achieved through increased plant density, pod-set at the plant and phytomer levels will decrease, but yield will increase to an upper limit that varies with environment (Manson *et al.*, 2024b). The special case of decoupling between faba bean growth and yield in some, but not all, high-growth environments merits further investigation (Manson *et al.*, 2025). Self-shading, light quality, and photoperiod could be involved (Nico *et al.*, 2015, 2016; Dreccer *et al.*, 2022; Quijano and Morandi, 2023). Where favourable conditions during the critical period are frequent, pollen supplementation or autofertility might alleviate pollen limitation if pollinator-dependent genotypes are the current standard.

## Conclusion

Variation in pod-set is explained by variation in resource availability and growth, not flower:pod ratio or putative competition between pods or between pods and shoots. Future research should begin by explaining pod-set as a function of critical period growth, then look for additional, smaller sources of variation that might complement growth to increase yield. Podding nodes are not modular, highlighting the need to address pod-set at plant and crop scales. Management should aim to maximize growth at the canopy level through appropriate genetics, sowing dates, and plant densities. For breeding, semi-dwarfism might retain the necessary plasticity in nodes per stem to capture environmental variation but shift allocation of growth from stems to yield. Variation in determinacy and sensitivity of shoots to fruit-set might be useful to match post-flowering phenology and growth to environment.

## Supplementary data

The following supplementary data are available at *JXB* online.

Fig. S1. Canopy versus plant yield in response to canopy thinning; data from Study 2.

Fig. S2, S3, and S4. Differences in individual, mean, and median pod profiles; data from Study 2.

Table S1. Wald test results from Study 2 showing that pods per node is a proxy for seed yield per node.

Table S2. Data sources and descriptions of experiments for pod profile data used in Study 3.

Table S3. Data sources and descriptions of experiments for determinacy data used in Study 4.

Table S4. Pod profile traits for Fig. 4.

Table S5. Pod profile traits for Fig. 5.

Table S6. Confidence intervals for reduced major axis regression coefficients of Fig. 10.

eraf176_suppl_Supplementary_Figures_S1-S3_Tables_S2-S6

## Data Availability

Study 1: yield, seed number and seed size data were provided by the GRDC by permission; data will be shared on request to the corresponding author with permission of the GRDC. Supporting seed number, size, biomass, and pod wall mass data that were analysed, but not generated, as part of this study are from previously published studies and datasets, and are cited within the text at relevant places. Study 2: the primary data supporting this study were not made publicly available at the time of publication. Study 3: supporting pod distribution data that were analysed, but not generated, as part of this review are from previously published studies and datasets, and are cited within the text at relevant places. Study 4: supporting pod distribution data that were analysed, but not generated, as part of this review are from previously published studies and datasets, and are cited within the text at relevant places.
