# Supplementary material for: Linking pod-set and seed yield of faba bean across organ, phytomer, plant, and population scales
Source: J Exp Bot. 2025 May 4;76(16):4472–89. doi: 10.1093/jxb/eraf176 (PMC12509879; doi:10.1093/jxb/eraf176)
Supplement: eraf176_suppl_Supplementary_Figures_S1-S3_Tables_S2-S6 [file eraf176_suppl_supplementary_figures_s1-s3_tables_s2-s6.pdf]

Supplementary Data

Manson et al. 2025, 'Linking pod-set and seed yield of faba bean across organ, phytomer, plant and population scales', Journal of Experimental Botany

1. Crop vs plant yield response to canopy thinning

In Experiments I, II and III, stand thinning increased plant yield up to 300 %, and the increase was proportional to the fraction of the growing season for which plants grew with more space (Figure S1). Despite this large response of individual plants, crop yield of thinned stands failed to match the un-thinned controls in 15 out of 16 cases; the exception was Nanu thinned at flowering in Experiment I.

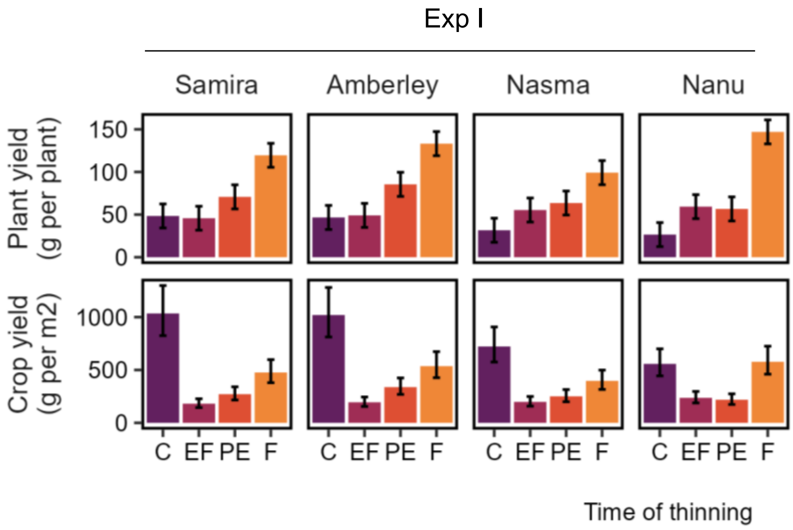

Figure S1. Plant and crop yield response to stand thinning, from ~23 plants m^{-2} to 4 plants m^{-2}, at several developmental stages in three experiments. C is un-thinned control, and timing of thinning is: EF, end of flowering; PE, pod emergence; P, podding; F, flowering. Error bars show the 95% confidence interval of the predicted mean. Data from Study 3.

2. Individual versus group pod profiles

Faba bean pod profiles in the literature are skewed towards basal, older nodes. However, these profiles use the average of a sample for pod number per node, while in our Experiments I, II and III the pod profile of individual plants was symmetrical (Figures S2, S3 and S4). Median pod number reflected the symmetrical distribution of individual pod number. It may be that, at the plant-level, pods on podding nodes collectively inhibit pod-set at nodes above the podding region.

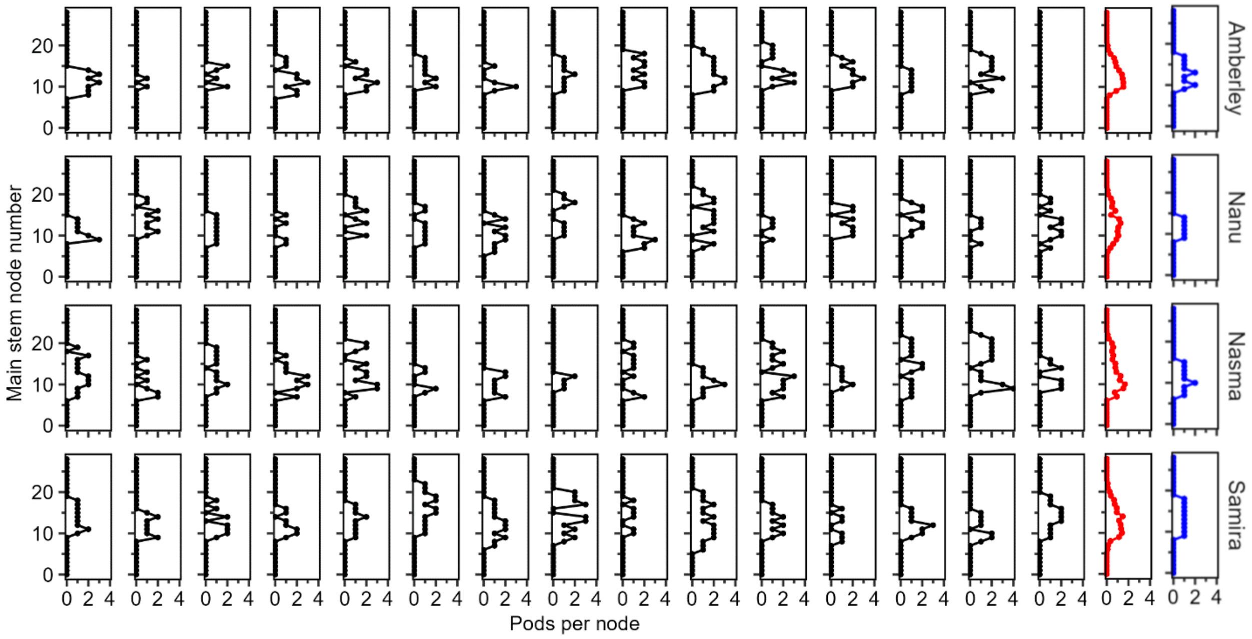

Figure S2. Profiles of pods per node for individual plants (black), and the sample average (by row, red) or median (blue). Data from control treatments of four genotypes (rows) in Experiment I (rainfed, 3 replicates, 5 plants per replicate).

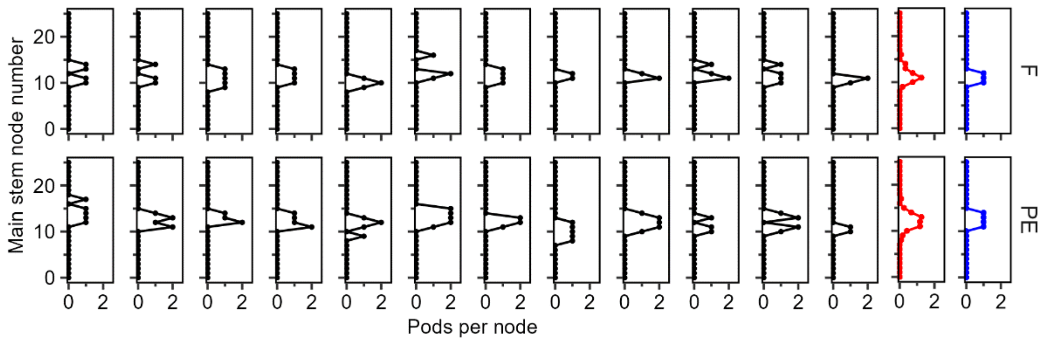

Figure S3. Profiles of pods per node for individual plants (black), and the sample average (by row, red) or median (blue). Data from control treatments of two timings (rows, F is control for treatments at flowering, PE is control for treatments at pod emergence) in Experiment II (irrigated, 3 replicates, 4 plants per replicate).

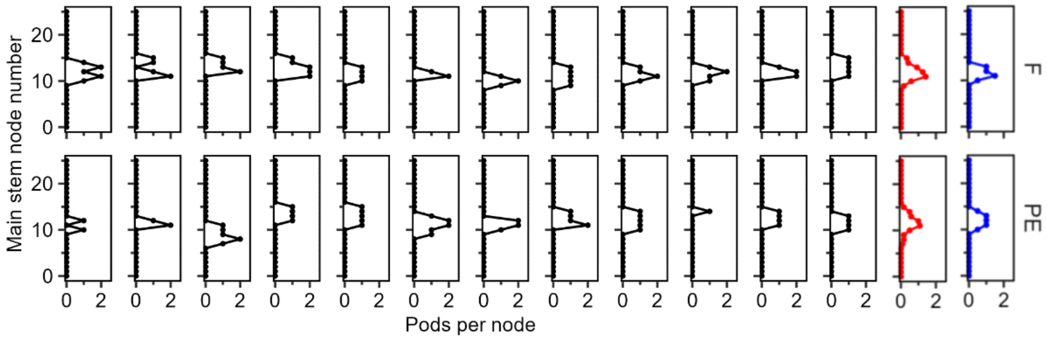

Figure S4. Profiles of pods per node for individual plants (black), and the sample average (by row, red) or median (blue). Data from control treatments of two timings (rows, F is control for treatments at flowering, PE is control for treatments at pod emergence) in Experiment III (rainfed, 3 replicates, 4 plants per replicate).

3. Pods per node as a proxy for seed yield per node

Pod number was an acceptable proxy for seed yield at the node level in Experiments I, II and III. Least-squares regression indicated little bias (Table S1), and R^2 was 0.91 or greater. Wald tests on the linear mixed models showed that there was no interaction between pod number and node position for seed yield per node; that is, pod number predicted seed yield consistently across nodes. Given similarly high associations in the literature (Adisarwanto and Knight, 1997; López-Bellido *et al.*, 2005) we concluded that pods per node is a widely reliable proxy for yield distribution along a faba bean stem.

Table S1. The association of seed yield per node (g node^{-1}) with pods per node, tested with least-squares regression (slope and intercept ± s.e., R^2) and Wald tests on linear mixed models (bold indicates p < 0.05).

	**Least-squares regression**			**Genotype**	**Wald test *p*-value**		
	Intercept	***Slope***	***R^2***		***Pod number***	***Node position***	***Pod:Node***
Exp I	-0.18 ± 0.06	2.01 ± 0.05	0.91	Amberley	**<0.001**	**0.024**	0.168
				Samira	**<0.001**	0.087	0.127
				Nanu	**<0.001**	0.106	0.325
				Nasma	**<0.001**	**0.006**	0.063
Exp II	-0.07 ± 0.03	1.76 ± 0.03	0.95	Samira	**<0.001**	**<0.001**	0.353
Exp III	-0.05 ± 0.03	1.63 ± 0.04	0.93	Samira	**<0.001**	**<0.001**	0.491

4. Choice of scale for pod profiles of thinning treatments

For thinning treatments in Experiments I, II and III, we were interested in comparing the effect of thinning on node-level pod-set *relative to other pods in the podding region*. We favoured the use of node position relative to the first podded node because 1) it focussed the analysis to our research question, 2) it reduced experimental noise between plants that varied in first podded node, and 3) we did not have node of first flower data for Experiment I, the scale we chose for comparing profiles after fruit removal treatments. Here, all nodes were counted from the first true leaf.

Statistical tests suggested that using node position from first podded node did not introduce bias to our comparisons of thinning treatments. In Experiment I, the node number of the first podded node was affected by genotype (p = 0.003), but not by thinning (p = 0.442) or their interaction (p = 0.187). The average first podded node was approximately node 8 for Nasma and Nanu, and node 9 for Amberley and Samira. In Experiments II and III (Samira only), first podded node was affected by fruit removal (p <0.001), as expected, but not by thinning, timing of treatment, or their interactions (p > 0.05). This shows that variation in first podded node was not confounded with thinning treatments, allowing comparison of profiles according to this scale. Standard errors of each treatment were 0.5 to 1.0 nodes, a range with little biological significance.

5. Data sources for pod profiles

Table S2. Data sources for pod profiles of Study 3.

Source	Country	Variety	Environmental variation	Plant density (plants m^{-2})
Adisarwanto & Knight 1997	Australia	Fiord	Three sowing dates	20, 39, 54
Grashoff 1990	Netherlands (Droevendaal)	Alfred	Two years x two irrigation treatments*	30
Loss et al. 1997	Australia	Fiord	Four sowing dates	30
Manson (Study 2, this publication)	Australia	Samira	Two location-years, irrigated / not irrigated in one location-year	24
Rowland et al. 1984	France, Canada	Strube, 370	Two location-years	NA
Stoddard 1993	Australia	Fiord	Four sowing dates	40
Aufhammer & Götz-Lee 1991	Germany	Herz Freya and Kleine Thuringer (combined)	No shade, Shaded flowering to end of flowering, Shaded flowering to maturity	20, 40, 70
Hodgson & Blackman 1956 (Experiment 2 and 4)	United Kingdom	Autumn faba from Rugby and Spring faba from Huntington	NA	11, 22, 39, 55
Ishag 1973	United Kingdom	Albyn Tick, Herz Freya, Maris Bead	NA	39, 64, 20, 52, 30, 57
Sprent et al. 1975	United Kingdom	Maris Bead	Control, Early shade, Late shade	19, 66
Hebblethwaite et al. 1984	United Kingdom	Tarvin, Herz Freya	Irrigated / Not irrigated	40
Stoddard 1986c	United Kingdom	L906	Field, Caged no bees	
Hebblethwaite et al. 1984	United Kingdom	Tarvin, Herz Freya	Caged with bees, Caged no bees	
Bishop et al. 2020	United Kingdom	Vertigo, Fury, Fuego	(2018 field trials) Caged no bees, Caged and tripped	
Kyllönen 2018	Finland	Kontu	Caged no bees, Caged with bees	

*With and without irrigation; this experiment included some treatments that were not appropriate for this study because they manipulated the distribution of water supply beyond the normal range of field conditions.

6. Data sources for determinacy

Table S3. Data sources for comparisons of indeterminate and determinate genotypes in Study 4.

Source	Country	Determinate variety	Indeterminate variety	Treatments
De Costa et al. 1997	United Kingdom	Tina	Gobo	Two years x Four irrigation treatments
Lateef & Azab 2017	Egypt	FLIP-87-117	Giza 461	Two years x five densities
Pilbeam et al. 1989	United Kingdom	Ticol	Alfred	Two years x 3 sowing dates
Pilbeam et al. 1990a	United Kingdom, France	Ticol, TP667	Maris Bead, M51	Five densities
Pilbeam et al. 1990b	United Kingdom	858	Bourdon	Year
Pilbeam et al. 1991	United Kingdom	858	Bourdon	Three years x five to six densities
Pilbeam et al. 1992	United Kingdom	Ticol	Minica	Two years x Five irrigation treatments
Silim & Saxena 1992	Syria	FLIP84230	ILB1814	Three years x two densities

7. Confidence intervals for RMA regressions of Figure 11

Table S4. Intercept and slope with confidence intervals for RMA regressions in Figure 11.

	Intercept	Slope
Stems per plant	0.11 [-0.29, 0.47]	1.33 [1.11,1.57]
Total biomass	-164 [-400, 26]	0.99 [0.81, 1.22]
Harvest index	0.09 [-0.03, 0.19]	0.73 [0.51, 1.00]
Seed yield	-12 [-70, 40]	0.80 [0.69, 0.92]

8. Pod profile traits for Figures 4 and 5

Table S5. Pod profile traits of Experiments II and III (Study 2) corresponding to Figure 4 in main text. Node numbers are relative to first flowered node at flowering.

Exp.	Treatment	First podded node	Last podded node	Podding duration	Node of max pod-set	Skewness	Maximum pod-set (s.e.)
II	Control stand, undamaged at flowering	1	7	7	2	0.29	1.00 (0.07)
	Control stand, fruits removed at flowering	5	9	5	6	0.40	1.80 (0.13)
	Control stand, undamaged at podding	1	9	8	5	0.63	1.25 (0.09)
	Control stand, fruits removed at podding	5	9	5	6	0.40	1.00 (0.07)
	Thinned stand, undamaged at flowering	1	8	8	3	0.38	2.50 (0.18)
	Thinned stand, fruits removed at flowering	5	10	5	5	0.00	3.04 (0.22)
	Thinned stand, undamaged at podding	1	6	6	3	0.50	1.75 (0.12)
	Thinned stand, fruits removed at podding	5	9	5	6	0.20	0.86 (0.06)
III	Control stand, undamaged at flowering	1	7	7	2	0.29	1.47 (0.18)
	Control stand, fruits removed at flowering	5	10	6	7	0.50	1.80 (0.22)
	Control stand, undamaged at podding	1	6	6	2	0.33	1.10 (0.13)
	Control stand, fruits removed at podding	5	8	4	6	0.50	0.7 (0.08)
	Thinned stand, undamaged at flowering	1	8	8	2	0.25	1.07 (0.13)
	Thinned stand, fruits removed at flowering	5	10	6	6	0.33	2.32 (0.28)
	Thinned stand, undamaged at podding	1	6	6	1	0.17	1.40 (0.17)
	Thinned stand, fruits removed at podding	5	8	4	6	0.50	0.51 (0.06)

Table S6. Pod profile traits of Experiments I, II and III (Study 2) corresponding to Figure 5 in main text. Node numbers are relative to first podded node at maturity.

Exp.	Treatment	First podded node	Last podded node	Podding duration	Node of max pod-set	Skewness	Maximum pod-set (s.e.)
I	Control stand	1	16	16	1	0.06	1.35 (0.07)
	Stand thinned at flower emergence	1	16	16	4	0.25	2.49 (0.12)
	Stand thinned at pod emergence	1	15	15	6	0.40	1.89 (0.09)
	Stand thinned at end of flowering	1	14	14	3	0.21	1.79 (0.09)
II	Control stand, undamaged at flowering	1	6	6	2	0.33	1.25 (0.12)
	Control stand, fruits removed at flowering	1	4	4	1	0.25	1.91 (0.18)
	Control stand, undamaged at podding	1	6	6	1	0.17	1.22 (0.12)
	Control stand, fruits removed at podding	1	4	4	1	0.25	1.07 (0.10)
	Thinned stand, undamaged at flowering	1	7	7	2	0.29	2.34 (0.22)
	Thinned stand, fruits removed at flowering	1	6	6	1	0.17	2.90 (0.28)
	Thinned stand, undamaged at podding	1	6	6	2	0.33	1.56 (0.15)
	Thinned stand, fruits removed at podding	1	5	5	1	0.20	1.31 (0.12)
III	Control stand, undamaged at flowering	1	5	5	2	0.40	1.42 (0.12)
	Control stand, fruits removed at flowering	1	3	3	1	0.33	2.32 (0.20)
	Control stand, undamaged at podding	1	5	5	1	0.20	1.17 (0.10)
	Control stand, fruits removed at podding	1	3	3	1	0.33	0.63 (0.05)
	Thinned stand, undamaged at flowering	1	6	6	3	0.50	2.14 (0.23)
	Thinned stand, fruits removed at flowering	1	5	5	1	0.20	2.17 (0.19)
	Thinned stand, undamaged at podding	1	5	5	1	0.20	1.31 (0.11)
	Thinned stand, fruits removed at podding	1	4	1	1	0.25	0.49 (0.04)
